# Prospective evaluation of quality of life effects in patients undergoing palliative radiotherapy for brain metastases

**DOI:** 10.1186/1471-2407-12-283

**Published:** 2012-07-10

**Authors:** Diana Steinmann, Yvonne Paelecke-Habermann, Hans Geinitz, Raimund Aschoff, Anja Bayerl, Tobias Bölling, Elisabeth Bosch, Frank Bruns, Ute Eichenseder-Seiss, Johanna Gerstein, Nadine Gharbi, Juliane Hagg, Matthias Hipp, Irmgard Kleff, Axel Müller, Christof Schäfer, Ursula Schleicher, Susanne Sehlen, Marilena Theodorou, Hans-Joachim Wypior, Franz Zehentmayr, Birgitt van Oorschot, Dirk Vordermark

**Affiliations:** 1 Radiation Oncology, Medical School Hannover, Hannover, Germany; 2 Radiation Oncology, Martin Luther University, Halle/Saale, Germany; 3 Radiation Oncology, TU München, München, Germany; 4 Radiation Oncology, St.-Josef-Hospital Gelsenkirchen-Horst, Gelsenkirchen, Germany; 5 Radiation Oncology Radiation Oncology, Hospital Krems, Krems, Austria; 6 Radiation Oncology, University of Münster, Münster, Germany; 7 Radiation Oncology, Hospital Düren, Düren, Germany; 8 Radiation Oncology, University of Frankfurt/Main, Frankfurt/Main, Germany; 9 Radiation Oncology, University of Ulm, Ulm, Germany; 10 Radiation Oncology, University of Regensburg, Regensburg, Germany; 11 Radiation Oncology, Hospital Traunstein, Traunstein, Germany; 12 Radiation Oncology, LMU München, München, Germany; 13 Radiation Oncology, Hospital Landshut, Landshut, Germany; 14 Radiation Oncology, Hospital Salzburg, Salzburg, Austria; 15 Radiation Oncology, University of Würzburg, Würzburg, Germany; 16 Radiation Oncology, Martin Luther University Halle-Wittenberg, Halle/Saale, Germany

**Keywords:** Brain tumours, EORTC-QLQ-C15-PAL, EORTC-BN20, Whole-brain radiotherapy

## Abstract

**Background:**

Recently published results of quality of life (QoL) studies indicated different outcomes of palliative radiotherapy for brain metastases. This prospective multi-center QoL study of patients with brain metastases was designed to investigate which QoL domains improve or worsen after palliative radiotherapy and which might provide prognostic information.

**Methods:**

From 01/2007-01/2009, n=151 patients with previously untreated brain metastases were recruited at 14 centers in Germany and Austria. Most patients (82 %) received whole-brain radiotherapy. QoL was measured with the EORTC-QLQ-C15-PAL and brain module BN20 before the start of radiotherapy and after 3 months.

**Results:**

At 3 months, 88/142 (62 %) survived. Nine patients were not able to be followed up. 62 patients (70.5 % of 3-month survivors) completed the second set of questionnaires. Three months after the start of radiotherapy QoL deteriorated significantly in the areas of global QoL, physical function, fatigue, nausea, pain, appetite loss, hair loss, drowsiness, motor dysfunction, communication deficit and weakness of legs. Although the use of corticosteroid at 3 months could be reduced compared to pre-treatment (63 % vs. 37 %), the score for headaches remained stable. Initial QoL at the start of treatment was better in those alive than in those deceased at 3 months, significantly for physical function, motor dysfunction and the symptom scales fatigue, pain, appetite loss and weakness of legs. In a multivariate model, lower Karnofsky performance score, higher age and higher pain ratings before radiotherapy were prognostic of 3-month survival.

**Conclusions:**

Moderate deterioration in several QoL domains was predominantly observed three months after start of palliative radiotherapy for brain metastases. Future studies will need to address the individual subjective benefit or burden from such treatment. Baseline QoL scores before palliative radiotherapy for brain metastases may contain prognostic information.

## Background

Quality of life (QoL) is now considered an important endpoint in oncological studies and is essential for the assessment of different therapeutic options. Knowledge about health-related QoL could help physicians, patients and even family members to achieve a better understanding of the treatment outcomes of cancer patients and make appropriate decisions. In the literature controversial results of measurement of QoL in patients with brain metastases have been reported [1-5]. Differences may have been caused by diverse points of time of assessment and the variety of fractionation schemes and patient cohorts. These results led us to address prospectively the question of the development of QoL over time within three months after initiation of palliative radiotherapy for brain metastases.

Physicians often estimate survival of patients much more positively than is realistic, potentially resulting in aggressive therapies without benefit [6,7]. The most commonly used instrument to estimate survival objectively in patients with brain metastases is the recursive partitioning analysis (RPA) [8,9] which relates overall survival after whole-brain radiotherapy (WBRT) to Karnofsky performance status (KPS), extracranial tumour status and age [10,11]. The more recently proposed graded prognostic assessment (GPA) included the number of brain metastases [12]. Rades et al. published a new scoring system to predict the survival of these patients treated with WBRT [11] and added the interval from tumour diagnosis to WBRT (longer time is better) to the criteria of RPA. They suggested an approach in which more favourable patients could be treated with longer-course WBRT to reduce neurotoxicity and other patients with poor prognosis could benefit from short-course WBRT.

Results of our prospective quality-of-life pilot study [1] indicated, that the initial QoL at start of radiotherapy was better in patients found to be alive at 3 months than in those deceased within 3 months. We therefore hypothesized that QoL at initiation of radiotherapy, may be an independent prognostic factor for survival of patients with brain metastases. Additionally, we observed a worsening of QoL in most domains in the pilot study, significant in drowsiness, hair loss and weakness of legs, during the first three months after the start of palliative radiotherapy for brain metastases. Other studies showed similar results with statistically significant deteriorations in fatigue [3], drowsiness and appetite [5] after the delivery of palliative radiotherapy for brain metastases.

Because our pilot study was limited by the small number of only n = 17 patients providing QoL data at the 3-month time point, we performed a follow-up large-scale study to address the following questions:

Which pre-treatment QoL domains and symptom scales increased or decreased significantly after 3 months? Which clinical characteristics influence the QoL after 3 months? What could be a benefit of radiotherapy?

Which pre-treatment QoL scales of the 3-month-survivors are significantly different from those of the non-survivors and could be a prognostic factor?

## Methods

### Recruitment

Patients with previously untreated brain metastases of solid tumours were recruited at 14 radiation oncology centres. Exclusion criteria were language barriers, insufficient compliance or cognitive status and chemotherapy during planned radiotherapy. After informed consent questionnaires EORTC QLQ-C15-PAL and BN20 were handed out to patients at the time of the initial consultation for the planned palliative brain radiotherapy. Patient and treatment characteristics, KPS and Barthel Index [13], a scale summarizing the ability to perform activities of daily living, were collected using patient records and documented follow up. Three months after the first radiotherapy session, survival status of patients was determined. We called patients by telephone and asked about their situation and performance. After that (if it was ethical), we sent out questionnaires with a stamped self-addressed envelope. If patients came for follow-up visits at the clinic, we also used this as an opportunity to hand out the questionnaire.

Ethics approval was obtained from the ethics committee at the University of Würzburg, Germany.

### Quality-of-life questionnaires

QLQ-C15-PAL and BN20 validated instruments were developed by the European Organization for Research and Treatment of Cancer (EORTC) Quality of Life Study Group for measuring the QoL of cancer patients in clinical trials [14]. QLQ-C15-PAL is a shortened form of QLQ-C30 for use in a palliative care setting, containing 15 items for the following nine domains: physical function, emotional function, global QoL, pain, fatigue, appetite, dyspnea, constipation and sleep. Each item is scored from 1 to 4 (″not at all″: 1; ″a little″: 2; ″quite a bit″: 3; ″very much″:4). As an exception, global QoL is scored from 1 (″very poor″) to 7 (″excellent″). The results for these domains are directly comparable between QLQ-C30 and QLQ-C15-PAL [15].

The BN20 questionnaire is a brain-specific module to be used in conjunction with the generic EORTC questionnaires and contains 20 items grouped into four domains (future uncertainty – four items, visual disorder, motor dysfunction and communication deficit – each three items) as well as seven single items (headaches, seizures, drowsiness, hair loss, itchy skin, weakness of legs, bladder control).

Questionnaire data were processed according to the procedures outlined in the EORTC QLQ-C30 scoring manual and the addendum for scoring of QLQ-C15-PAL [15,16]. For functional scales and global QoL, high scores represent good functioning/ good QoL. For the symptom scales and for all scales of BN20, high scores indicate severe symptoms.

We asked proxies of patients to simultaneously participate in a separate questionnaire to report the QoL of their relative. Results of the proxy assessment in the main study will be published separately.

### Statistical analysis

The ratings of each item of both questionnaires were linearly transformed to a 0–100 scale, with ‘not at all’ conforming to 0 and ‘very much’ conforming to 100 [15]. QoL scores were then analyzed parametrically [17]. Mean change in scores about 10 to 20 (“moderate”) and greater than 20 (“worse”) were considered clinically relevant [18].

Means, mean differences, SEs, test statistics and p-values for all QoL subscales, separate for time points and patient groups, were calculated.

Paired t-tests were used to compare the patients´ mean scores between the points in time. For each scale, a multiple regression analysis was used to find the best prediction equation for the change of QoL ratings after three months. The following variables were included in the analysis: pre-therapeutic KPS, age, number of cerebral metastases [1–3 vs. >3], therapeutic strategy [WBRT vs. SRS/hfSRT], localization [breast, lung, other], and pre-therapeutic status of the primary tumour [progressive vs. non-progressive]. Dummy variables were created for all nominal variables with more than two categories. Variables without significant influence were excluded backwards. This method avoids insignificant predictors and can exclude intercorrelated predictors.

Student’s t-tests for independent samples were used to test differences in the QLQ scales between 3-month-survivors and non-survivors. A multiple logistic regression model was used to test which variables can significantly predict 3-month survival. The QoL scales with significant change in the pilot study (physical function, fatigue, pain, motor dysfunction, weakness of legs, communication deficit and headaches), and the mentioned non-QoL independent variables (number of brain metastases, extracranial primary tumour situation or extracranial metastases, KPS, age, primary tumour) were included in the analysis. Variables without significant influence were excluded stepwise backwards.

The significance level was set to .05. For further explorative analyzes, the significance level was set to .01 to adjust for multiple comparisons (several scales and groups).

## Results

### Patients and treatment characteristics

Including patients of the pilot study (n = 64) [1] 151 patients with previously untreated brain metastases were recruited at 14 centres in Germany and Austria from 01/07 to 01/09. Most patients were recruited in only few (6) centres with 10 to 50 patients. Other centres were only small and recruited only few patients with brain metastases. The dominant radiotherapy strategy (n = 131, 88.5 %) was whole-brain radiotherapy (WBRT) alone. Eight patients additionally received boost irradiation, 17 (11.5 %) patients stereotactic radiosurgery (SRS), hypofractionated stereotactic radiotherapy (hfSRT) or 3D-conformal local radiotherapy alone.

Patient and treatment characteristics, including the pre-treatment KPS and the Barthel index are presented in Table 1. The survival status was known in 142 patients, 54 (38 %) had died within three months (until 90 days) after beginning of radiation therapy. Median overall survival was 135 days (4.5 months), 5.6 months for patients with limited (1–3) brain metastases and 3.4 months of patients with multiple brain metastases.

**Table 1:** Baseline clinical and treatment characteristics of patients registered for prospective QoL assessment

**age [years]**	**median (range)**	**61 (33–84)**	
	**total**	**n = 151**	**100 %**
primary tumor	non-small cell lung cancer	70	46
	small-cell lung cancer	20	13
	melanoma	12	8
	renal cell carcinoma	7	5
	colorectal cancer	3	2
	breast cancer	25	17
	others	12	8
	unknown	2	1
Karnofsky performance score	≥ 70	119	79
	< 70	30	20
	unknown	2	1
RPA classification	1	17	11
	2	99	66
	3	33	22
	unknown	2	1
GPA classification	0-1.0	63	43
	1.5-2.5	70	48
	3.0	11	7
	3.5-4.0	3	2
	unknown	4	3
Barthel index	90-100	115	76
	<90	34	23
	unknown	2	1
fractionation of whole-brain radiotherapy	10x3 Gy	80	53
	14x2.5 Gy	12	8
	others	32	21
	unknown	5	3
fractionation of stereotactic radiotherapy	1x18-20 Gy	10	7
	7x5 Gy	4	3
	5x6 Gy	4	3
	10x4 Gy	1	1
initial steroid dose (prednisone equivalent)	no steroids	41	27
	daily dose <50 mg	38	25
	daily dose 50–100 mg	38	25
	daily dose >100 mg	26	17
	unknown	8	5
extracranial tumor status	primary tumor (PT) not detectable	54	36
	PT detectable, not progressive	44	29
	PT progressive	48	32
	PT status unknown	5	3
	any extracranial metastases	104	70
intracranial tumor status	>3 metastases	83	56
	1-3 metastases	65	44
	number of metastases unknown	4	3
	largest metastasis >2 cm	53	35
	diameter of metastases unknown	17	12

### Completion of questionnaires

All 151 patients completed baseline QoL questionnaires EORTC QLQ-C15-PAL; 149 patients the BN20. 66 patients completed questionnaires at both time points. 53 of them were returned after 3 months (range 60–120 days from initiation of radiotherapy). One patient filled in the second questionnaire 70 days after start of radiotherapy and died on day 87. The response rate of 3-month survivors (>90 days) was 62 of 88 (70.5 %).

### QoL after 3 months in comparison to pre-treatment

Self-assessed QoL was compared for the time points before and three months after the start of radiotherapy, including only data from patients who completed questionnaires both at the start of radiotherapy and after 3 months, to eliminate a potential bias resulting from a selection of survivors at the latter point of time.

**Figure 1:**
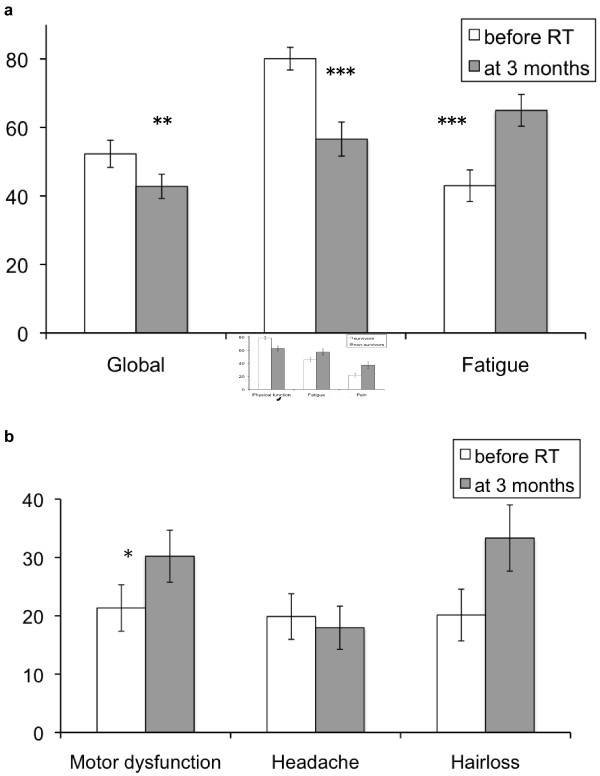
**Self-assessed QoL in preselected QLQ scales before and 3 months after start of RT. a**) EORTC QLQ C15-PAL: global QoL, physical function (higher score better), and fatigue (higher score worse). **b**) EORTC BN20: motor dysfunction, headaches and hairloss (all higher score worse) (*p < .05, ** p < .01, ***p < .001; paired-t-test, one-tailed).

Explorative analysis regarding the QLQ-C15-PAL scales revealed a significant deterioration in global QoL (p = 0.011), physical function (p < 0.001), fatigue (p < 0.001), pain (p = 0.034), nausea (p < 0.001), and appetite loss (p < 0.001). In the organ-specific BN20 module, a significant deterioration in drowsiness (p < 0.001), motor dysfunction (p = 0.014), communication deficit (p = 0.025) and weakness of legs (p < 0.001) was noted, whereas a trend was observed for increased hair loss (p = 0.068) and the score for headache remained unchanged (Figures 1a-b).

Steroid use of 3 months-survivors was significantly lower at the second point of time (63 % vs. 37 %, p = 0.002).

The therapeutic strategy (WBRT only: worse function, p = 0.041), and the localization of the primary tumour (breast: better function, p = 0.025) were significantly associated with the physical function score after three months. The therapeutic strategy (WBRT worse than SRS/hfSRT) was significantly associated with the fatigue score after three months (p = 0.001).

The initial KPS score was significantly associated with the motor dysfunction (p = 0.004).

In several QoL domains, the baseline scores before treatment were significant predictors for the scores of the same domains after three months (global QoL p < 0.001, physical function p = 0.006, fatigue p < 0.001, motor dysfunction p = 0.001, headache p = 0.036).

### Baseline QoL as prognostic factor for survival

**Figure 2:**
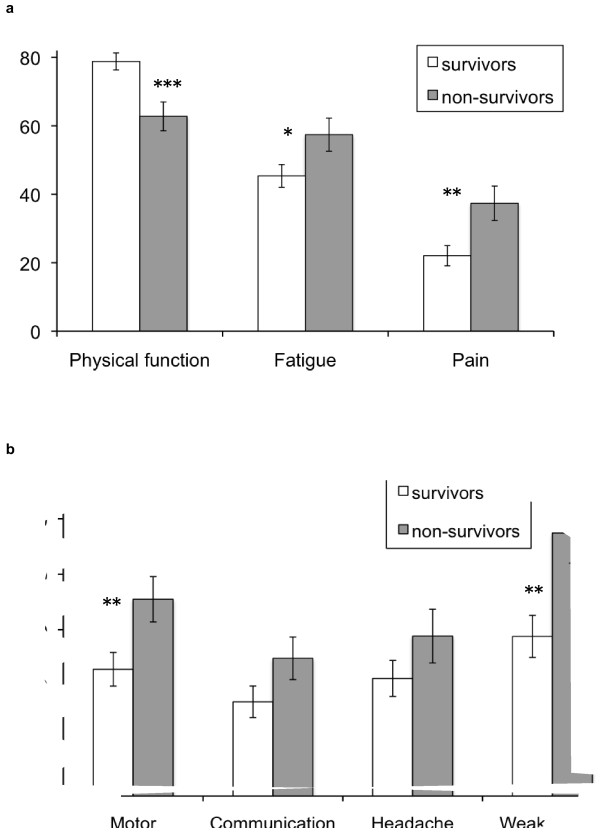
**Differences in pre-treatment QoL between 3-month surviviors vs. non-survivors. a** )EORTC QLQ-C15-PAL: physical function (higher score better), fatigue and pain (both: higher score worse). **b**) EORTC BN20: motor dysfunction, headaches and hairloss (higher score worse) (*p < .05, ** p < .01, ***p < .001; paired-t-test, one-tailed).

Baseline pre-treatment scores of physical function (p = 0.001), fatigue (p = 0.035), pain (p = 0.01), motor dysfunction (p = 0.014), and weakness of legs (p = 0.006) were significantly better in 3-month survivors than in non-survivors (Figure 2a-b). However, no significant differences were detected in the remaining preselected domains of communication deficit and headache. Explorative analysis regarding the other QLQ-C15 PAL/BN20 scales revealed a significant difference at baseline between 3-month survivors and non-survivors in the symptom scale of appetite loss (p < 0.01).

Using a multiple logistic stepwise regression model we found three significant predictors of 3-month survival. Patients with higher age (p = 0.04), lower KPS scores (p = 0.001), and higher pain scores (QLQ-C15-PAL score, p = 0.04) were found more frequently in the group of non-survivors.

## Discussion

This prospective study generated a large QoL data set related to the radiotherapy of brain metastases with a validated brain-specific QoL questionnaire. Only one study group reported on a bigger patient group of 170, but used a general QoL tool (ESAS – Edmonton Symptom Assessment Scale) without a brain-specific module [5].

Three smaller trials used the Functional Assessment of Cancer therapy – General scale (FACT-G) with a brain module (FACT-BR) [19-21]. The results are not comparable to our study because of the addition of temozolomide to WBRT [19] or different evaluation points of time. The brain module BN20 has been validated by the EORTC in an international study incorporating 891 patients with primary brain tumours [22] and has been applied in some smaller QoL studies of patients with brain metastases [4,23,24]. Because of short assessment periods [4,15] after radiotherapy or analysis only of special radiation techniques like radiosurgery these studies are not directly acceptable to the routine palliative radiotherapy setting with predominant whole-brain radiotherapy and short survival times.

The BN20 questionnaire was used in this study with the shortened questionnaire variant EORTC QLQ-C15-PAL to reduce the burden of repeated questionnaire completion for the incurable patients. It should be noted that the short version QLQ-C15-PAL lacks the two questions regarding cognitive function from QLQ-C30 [15], and therefore some potentially interesting information was lost by the decision for the shorter version.

QoL was evaluated at only two points in time. The second point of time after 3 months was chosen to reduce the effects of rapid deterioration by very early tumour progression, while maintaining a reasonable number of patients available for assessment. It must be acknowledged that the addition of both earlier and later points of time would have been informative. However, a distinction between brain-metastasis-related, extracranial-tumour-related and treatment-related impairment would probably have been similarly problematic at other points of time. The choice of the 3-month-point was a compromise for pragmatic reasons. Given all the problems associated with the highly palliative situation of the patients studied, a response rate of 70 % of survivors at this point of time appears adequate.

The overall survival in the patient group (38 % dead at three months) is comparable to other reports [3]. Previously, improved survival in patients with brain metastases has been linked to lower RPA class [10], higher GPA scores [25,26], with stable extracranial tumour situation, limited (1–3) intracranial metastases and therefore possible radiosurgery [27,28] and low steroid dose [29].

One of the most important results of this study was the deterioration of different domains and symptom scales of QoL in patients after three months. This was acceptable in potentially treatment-related symptom scales such as hair loss (only a trend in this study) and fatigue. In our study 42 % and 54.2 % of patients showed an increase of hair loss and fatigue scores, respectively, over 20 points. This can be considered clinically relevant. The study of Slotman et al. [17] examined QoL of patients with or without prophylactic cranial irradiation (PCI) for small-cell lung cancer with the same questionnaires. Slotman et al. [17] found a worsening hair loss (≥ 20 points) in 22.4 % of patients with PCI and 12.2 % of controls and a worsening of fatigue on this order of magnitude in 49 % of patients with PCI and 26.7 % of controls. Hair loss and fatigue are both also potential chemotherapy-associated symptoms. In our study subsequent chemotherapies after irradiation were not evaluated. Our findings are comparable with the results of Wong et al. who reported more severe fatigue symptoms in 57 % of patients over time [3]. The testing of influencing factors in our study showed a significant association of WBRT as treatment strategy and the fatigue score after three months.

The score for headache was slightly better after 3 months in the pilot phase of this study and remained unchanged in the now reported main phase with a larger cohort of patients. Additionally, the steroid use after 3 months was significantly lower. Therefore, these results show one important benefit of the radiotherapy.

The deterioration of global health status, physical function and of symptom scales like motor dysfunction, communication deficit or weakness of legs after three months were not necessarily expected. The most important aim of brain irradiation in patients with brain metastases should be the improvement or stabilization of the performance status and of QoL.

Intracranial and extracranial progression or adverse treatment effects are potential explanations for the deterioration of QoL within three months after start of radiotherapy. Further published QoL studies did not analyze intra- or extracranial progression over time. This study provided some limited information on progression of the primary, additional extracranial and intracranial metastases or increase in the size of brain metastases. Due to the poor condition of most patients, it was felt to be unethical to require specific imaging studies to be performed at defined points of time. Imaging data after radiotherapy was available for 53 % of patients and showed intracranial progression in 20 % and extracranial progression in 53 % of patients among these patients. For specific preselected QoL domains, predominant factors influencing the scores at the 3-month-point were identified. For instance, brain metastases from breast cancer were associated with better physical function at 3 months. This may be related to the known slightly better prognosis after radiotherapy for patients with brain metastases from breast cancer compared to other primaries [30] but also to the more recently documented improved outcome of subgroups e. g. with positive Her-2 status [31].

A known prognostic factor in brain metastases patients, KPS, was associated with motor dysfunction after three months. The therapeutic strategy (WBRT vs. stereotactic radiotherapy) was significantly associated with the physical function score during the same period. Although the limited information on imaging response of brain metastasis precludes definitive conclusions, factors related to the initial selection of patients for specific strategies (e. g. stereotactic radiotherapy for fitter patients with limited number of metastases) are likely to explain part of the variation in post-radiotherapy QoL. Data from the literature suggests that achieving local control of brain metastases is a prerequisite for maintaining neurologic function[32]. Therefore, patients treated with palliative whole-brain radiotherapy alone may deteriorate not because of, but despite of radiotherapy, not considering the frequent rapid extracranial progression of metastases.

A second main result of this study was the difference of baseline QoL of 3-month survivors vs. non-survivors. Baseline pre-treatment QoL scores of physical function, fatigue, pain, appetite loss, motor dysfunction, weakness of legs were significantly better in survivors suggesting that these scores might contain prognostic information.

Similar results were presented by Movsas et al. who analyzed the QoL of 239 patients with locoregionally advanced non-small-cell lung cancer treated with amifostine and chemotherapy using the EORTC QLQ-C30 and LC-13 (RTOG 9801) [33]. Patients with a global QoL score less than 66.7 had an approximately 70 % higher rate of death than patients with scores of ≥66.7 (p = 0.012). Other QoL predictors for OS were physical functioning and dyspnoea. In their study, these QoL scales seemed to be more relevant and powerful as prognostic factors than standard measures like KPS, so the authors suggested patient-reported QoL as a good stratification factor in future.

Other prior studies with lung cancer patients, who were also a predominant subgroup in the present cohort, have shown similiar results. Montezari et al. reported the initial global QoL as the most significant predictor of survival at 3 months [34]. In the literature, global QoL, appetite loss, fatigue and pain were the most important indicators for predicting survival times in cancer patients after adjusting known clinical prognostic factors [35,36]. It is argued that measures such as global QoL are patient-rated and thus have the potential to reflect the patient's well-being better than physician's observed indicators. Patient-reported outcomes detect prognostically relevant lowered patient well-being earlier than other measures. Higher scores correlate with more positive behaviour and reflect individual characteristics that affect survival [36]. Data for high-grade glioma patients defined fatigue as a prognostic factor [37] but no other trial has so far shown a specific QoL score of patients with brain metastases as predictive.

In the present study, using a multiple logistic stepwise regression model, only pain as one of six predetermined QoL domains, when tested together with clinical variables, remained prognostically significant. KPS and age were also significant predictors of survival. One possible explanation is that the KPS, as a global performance measure, contains information overlapping with that of the selected QoL domains. It could be argued that KPS alone integrates some of the prognostically relevant information obtainable from the QoL questionnaires. The prognostic role of pain, as assessed by EORTC QLQ-C15-PAL, for survival may be that on an indicator of uncontrolled extracranial disease. Sundaresan et al. developed a prognostic index for patients with WBRT for brain metastases of lung cancer and scored the factors age, ECOG performance status, histology, weight loss, primary and systemic disease status [38]. The new scoring system suggested by Rades et al. predicted the survival of all patients with brain metastases treated with WBRT [11]. Actuarial 6-month survival varied from 6 % of patients in the worst prognostic group, via 15 % and 43 % to 76 % of patients with better status. No QoL scores were used because of the retrospective nature of these studies. Sperduto et al. defined a disease-specific GPA, because patients with brain metastases are a heterogeneous population and different primaries are not comparable [26].

Potentially, the inclusion of self-reported QoL scores in prognostic scoring systems for patients with brain metastases may be more relevant within more specifically defined patients subgroups, e. g. only lung cancer or breast cancer patients. Given the generally unsatisfactory QoL outcomes after palliative radiotherapy for brain metastases, novel strategies to improve intracranial tumour control are needed. Further QoL studies are necessary to better identify patients groups who may benefit from specific modes of radiotherapy, e. g. shorter or longer-course WBRT, local (stereotactic) treatment alone or combinations. At the same time, a patient subgroup with very poor baseline characteristics (including clinical and QoL parameters) may be defined, in whom radiotherapy can be withheld.

## Conclusions

In a prospective study, QoL of patients with brain metastases deteriorated in several domains within three months of initiation of palliative radiotherapy. However, headache remained unchanged and steroid use decreased at 3 months compared to baseline. Although 3-month survivors differed in several areas of pre-treatment QoL scores from non-survivors, only the QoL score for pain was predictive of survival when clinical variables were also considered.

## Abbreviations

EORTC, European Organisation for Research and Treatment of Cancer; EORTC-QLQ-C15-PAL, Quality of life questionnaire of EORTC, shortened version with 15 questions for palliative setting; EORTC-BN20, Quality of life questionnaire of EORTC for patients with brain tumours; GPA, Graded Prognostic Assessment; Gy, Gray; hfSRT, Hypofractionated Stereotactic Radiotherapy; KPS, Karnofsky Performance Score; MeV, Mega electron Volts; QLQ, Quality of life questionnaire; QoL, Quality of life; RPA, Recursive Partitioning Analysis; RT, Radiotherapy; SRS, Stereotactic Radiosurgery; WBRT, Whole-brain Radiotherapy.

## Competing interests

The authors declare that they have no competing interests.

## Authors’ contributions

DV, SS, CS and HG participated in the design of the study. YP, DV and DS performed the statistical analyses. All authors provided study material and were involved in manuscript writing; they read and approved the final manuscript. DS, BO and DV drafted the manuscript.

## Author’s information

Dirk Vordermark on behalf of the Quality of Life Working Party of the German Radiation Oncology Society (DEGRO).

## Pre-publication history

The pre-publication history for this paper can be accessed here:

http://www.biomedcentral.com/1471-2407/12/283/prepub
